# Tumor-Associated Macrophages Recruit CCR6^+^ Regulatory T Cells and Promote the Development of Colorectal Cancer via Enhancing CCL20 Production in Mice

**DOI:** 10.1371/journal.pone.0019495

**Published:** 2011-04-29

**Authors:** Jinlin Liu, Ning Zhang, Qun Li, Weiwei Zhang, Fang Ke, Qibin Leng, Hong Wang, Jinfei Chen, Honglin Wang

**Affiliations:** 1 Shanghai Institute of Immunology, Institute of Medical Sciences, Shanghai Jiao Tong University School of Medicine, Shanghai, People's Republic of China; 2 Department of Pathology, Institute of Medical Sciences, Shanghai Jiao Tong University School of Medicine, Shanghai, People's Republic of China; 3 State Key Laboratory of Medical Genomics at Ruijin Hospital, Shanghai Jiao Tong University School of Medicine, Shanghai Institute of Hypertension, Shanghai Key Laboratory of Vascular Biology, Shanghai, People's Republic of China; 4 Institut Pasteur of Shanghai, Chinese Academy of Science, Shanghai, People's Republic of China; 5 Department of Oncology, the Affiliated Nanjing First Hospital, Nanjing Medical University, Nanjing, People's Republic of China; University of Muenster, Germany

## Abstract

**Background:**

Tumor-associated macrophages (TAMs) remodel the colorectal cancer (CRC) microenvironment. Yet, findings on the role of TAMs in CRC seem to be contradictory compared with other cancers. FoxP3^+^ regulatory T (Treg)-cells dominantly infiltrate CRC. However, the underlying molecular mechanism in which TAMs may contribute to the trafficking of Treg-cells to the tumor mass remains unknown.

**Methodology/Principal Findings:**

CRC was either induced by N-methyl-N-nitrosourea (MNU) and H. *pylori* or established by subcutaneous injection of mouse colorectal tumor cell line (CMT93) in mice. CMT93 cells were co-cultured with primary macrophages in a transwell apparatus. Recruitment of FoxP3 green fluorescence protein positive (FoxP3^GFP+^) Treg-cells was assessed using the IVIS Imaging System or immunofluorescence staining. A role for macrophages in trafficking of Treg-cells and in the development of CRC was investigated in CD11b diphtheria toxin receptor (CD11b-DTR) transgenic C57BL/6J mice in which macrophages can be selectively depleted. Treg-cells remarkably infiltrated solid tumor, and predominantly expressed the homing chemokine receptor (CCR) 6 in the induced CRC model. Both CMT93 cancer cells and macrophages produced a large amount of CCL20, the sole ligand of CCR6 *in vitro* and *in vivo*. Injection of recombinant mouse CCL20 into tumor sites promoted its development with a marked recruitment of Treg-cells in the graft CRC model. Conditional macrophage ablation decreased CCL20 levels, blocked Treg-cell recruitment and inhibited tumor growth in CD11b-DTR mice grafted with CMT93.

**Conclusions/Significance:**

TAMs recruit CCR6^+^ Treg-cells to tumor mass and promote its development via enhancing the production of CCL20 in a CRC mouse model.

## Introduction

Colorectal cancer (CRC) is one of the most common cancers worldwide, and the fourth most common cause of cancer-associated death [1]. Similar to other types of cancer, CRC arises in chronically inflamed tissue, and its development is under immune surveillance and depends on cytokines and chemokines secreted from tumor-infiltrating immune cells [2].

Regulatory T (Treg)-cells were initially characterized by their CD4^+^CD25^+^FoxP3^+^ phenotype and are thought to balance necessary aggressiveness against foes with tolerance for self-constituents [3]. Increased numbers of Treg-cells have been reported in the blood and tumors of patients with various cancers, including breast cancer, colorectal cancer, esophageal cancer, gastric cancer, hepatocellular carcinoma, leukemia, lung cancer, lymphoma, melanoma, ovarian cancer, and pancreatic cancer [4]. Moreover, studies that depleted Treg-cells resulted in improved antitumor immunity and markedly reduced tumor growth in several mouse tumor models [4]. In patients with CRC, an increased numbers of Treg-cells in peripheral blood, mesenteric lymph nodes and directly within the tumor were observed [5], [6]. Moreover, antibody-mediated FoxP3 protein therapy resulted in a clinically significant reduction in tumor burden in a syngeneic model of colon cancer metastasis to liver in Balb/c mice [7].

Knowledge regarding the origin of Treg-cells accumulating inside tumor lesions comes from animal tumor model studies. It has been proposed that both pre-existing naturally occurring Treg-cells (nTreg-cells) and adaptive Treg-cells (aTreg-cells) induced from non-Treg precursor cells contribute to the total pool of tumor-infiltrating FoxP3^+^ Treg-cells [8], [9]. Intratumoral Treg-cells are antigen experienced, highly activated and appear to undergo extensive cellular proliferation as the majority of intratumoral Treg-cells have lost CD62L and CD45RB expression, but their expression of CD25, CTLA-4 and GITR is strongly increased [8], [10]. The depletion of pre-existing nTreg-cells using anti-CD25 mAb resulted in more rapid and extensive conversion of precursor cells to aTreg-cells, which can largely compensate for the loss of nTreg-cells [8]. The activation and expansion of either nTreg-cells or aTreg-cells at tumor sites is antigen dependent, and contributes to the tumor specific tolerance [4]. In particular, antigen presenting cells such as tumor-infiltrating DCs and macrophages are likely involved in the local activation and expansion of nTreg-cells inside tumor lesions [11]. Moreover, in humans, tumor antigen specific FoxP3^+^ Treg-cells have been successfully isolated from tumor tissues as well as peripheral blood of patients with melanoma and cervical cancer [12], [13].

FoxP3^+^ Treg-cells begin to infiltrate into the incipient tumor lesion at a very early stage of tumor development [14]. Studies with human ovarian cancers have shown that Treg-cells traffick to the tumor mass and ascites via chemokine receptor CCR4 responding to CCL22 released by tumor cells and tumor-associated macrophages (TAMs). This suggests that chemokine and chemokine receptors are important for Treg-cell homing to tumours [15]. However, other chemokine receptors, for example, CXCR3, and CCR6, which are shared with memory Th1, Th2 and Th17 cells, have also been shown to be expressed by tumor-infiltrating Treg-cells [16], [17]. CCR6 is a chemokine receptor with only one single known specific chemokine ligand, the inflammatory and homeostatic chemokine CCL20 which is also known as liver and activation-regulated chemokine (LARC) [18] or Exodus [19]. In contrast to many other chemokines, CCL20 has a relatively specific receptor and binds almost exclusively to the chemokine receptor CCR6, which is also expressed by CD45RO^+^ memory T cells and others [20].

Despite the fact that tumor development could be promoted in part by the immune system itself, the suppressive function of Treg-cells, as well as the molecular mechanism underlying their trafficking to tumor sites remains elusive. In this study, we investigated the homing characteristics of Treg-cells, and the potential tumor-promoting function of TAMs in a CRC model. We report that, in solid tumors of mice with CRC, Treg-cells were significantly increased compared to control groups. TAMs contributed to the increased CCL20 production in the tumor environment and recruited Treg-cells that expressed high levels of CCR6. Injection of recombinant mouse CCL20 into CRC promoted tumor development with a marked recruitment of Treg-cells in wild-type mice, and stimulated tumor growth by directly activating CMT93 cells in CCR6−/− mice. Most importantly, using CD11b-DTR transgenic mouse model we demonstrate for the first time that conditional macrophage ablation not only decreased expression of CCL20 and FoxP3 but resulted in a significant inhibition of CRC. Our data suggest that selectively targeting CCL20 by down-regulating the activity of TAMs or its counter receptor CCR6 to block memory Treg-cell recruitment may represent a novel therapeutic strategy against CRC in humans.

## Results

### Numbers of FoxP3^+^ Treg-cells were increased in tumor-infiltrating lymphocytes of CRC in mice

**Figure 1:**
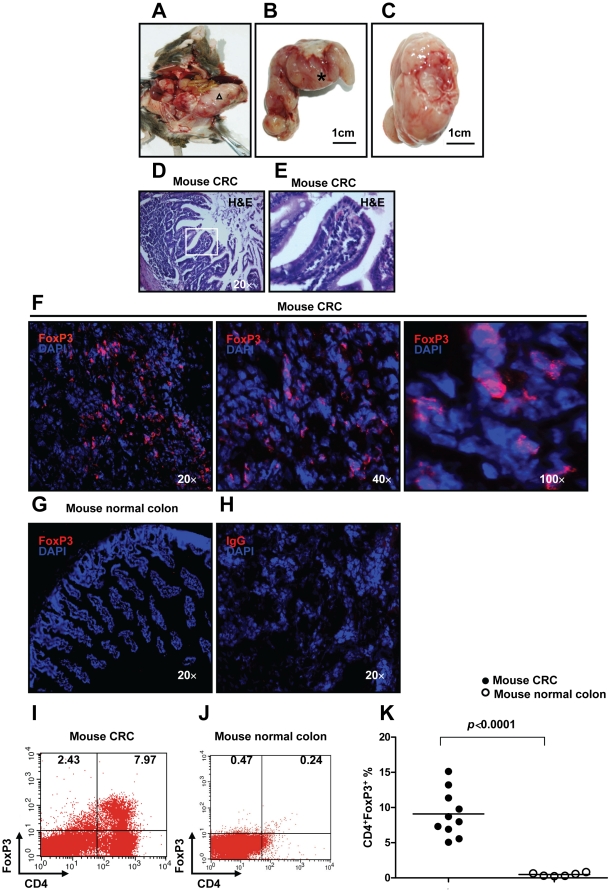
Increased numbers of FoxP3^+^ Treg-cells in tumor-infiltrating lymphocytes of CRC induced by MNU and H. *pylori*. in mice. (**A**) Eighty weeks old C57BL/6J mouse with large CRC induced by MNU and H. *pylori*. (**B**) Higher magnification of the area indicated by the triangle (Δ) of (A). (**C**) Transect of the area indicated by the asterisk (*) of (B). (**D**) H&E staining of colorectal carcinoma tissue derived from CRC induced by MNU and H. *pylori* (original magnification, ×20). (**E**) Higher magnification of (D) as indicated by the rectangle. To examine FoxP3^+^ Treg-cells in mice with CRC induced by MNU and H. *pylori*, immunofluorescence staining of FoxP3 was performed on cryosections from mouse CRC or normal colon tissue. (**F**) Expression of FoxP3 (Red staining) in mouse CRC (original magnification, ×20, ×40 and ×100). (**G**) Negative expression of FoxP3 in mouse colon tissue (original magnification, ×20). (**H**) Rat IgG control staining (original magnification, ×20). Lymphocytes infiltrating CRC (n = 10) and normal mucosa (n = 6) were isolated for Flow cytometric analysis of FoxP3 expression. (**I**, **J**) The frequency of Treg-cells in CRC derived from eighty weeks old C57BL/6J mouse and normal mucosa in mice. (**K**) A significant increase in the frequency of Treg-cells was found in TIL of CRC compared with normal mucosa (*p*<0.0001 by Student's t Test). Representative data are shown which had been reproduced in 3 independent experiments.

A major goal of the present study was to provide a definitive demonstration of the trafficking mechanism for FoxP3^+^ Treg-cells in CRC *in vivo*. To this end, we induced CRC in mice with MNU and H. *pylori*. MNU is a direct alkylating agent that does not require metabolic activation and thus is a potent topical carcinogen [21]. Previously, intrarectal instillation of MNU has been reported to induce CRC in rats [21]. As expected, after 80 weeks, 85% mice used in this study developed visible CRC induced by MNU and H. *pylori* (**Figure 1A–C**). H&E staining further showed scattered tumor cells in sections derived from CRC in mice (**Figure 1D, E**). These data clearly suggest that the method used in this study represents an effective strategy for the induction of CRC in mice.

To investigate whether FoxP3^+^ Treg-cells were infiltrating tumors induced by MNU and H. *pylori* in mice, immunofluorescence staining of the tumor and normal colon for FoxP3 was performed. As observed in PBMC of human patients (**Figure S1**), FoxP3 (Red staining) was abundantly expressed in tumor tissue derived from mice treated with MNU and H. *pylori* compared with normal colon of untreated mice or IgG control staining (**Figure 1F–H**). To look more precisely at the frequency of Treg-cells infiltrating the CRC, single cell suspension of CRC and normal mucosa was prepared and the frequency of Treg-cells was determined by Flow cytomery. We confirmed that a significantly higher frequency of CD4^+^FoxP3^+^ Treg-cells were present in the tumor, as compared to normal colon (mean 9.095%±1.038% vs mean 0.478%±0.095%, *p*<0.0001) (**Figure 1I–K**). The increased density of Treg-cells in tumor-infiltrating lymphocytes (TIL) strongly suggests that Treg-cells may play an essential role in modulating anti-tumor immune responses in the mouse model of CRC induced by MNU and H. *pylori*.

### CCR6 was required for recruitment of Treg-cells to CRC in mice

**Figure 2:**
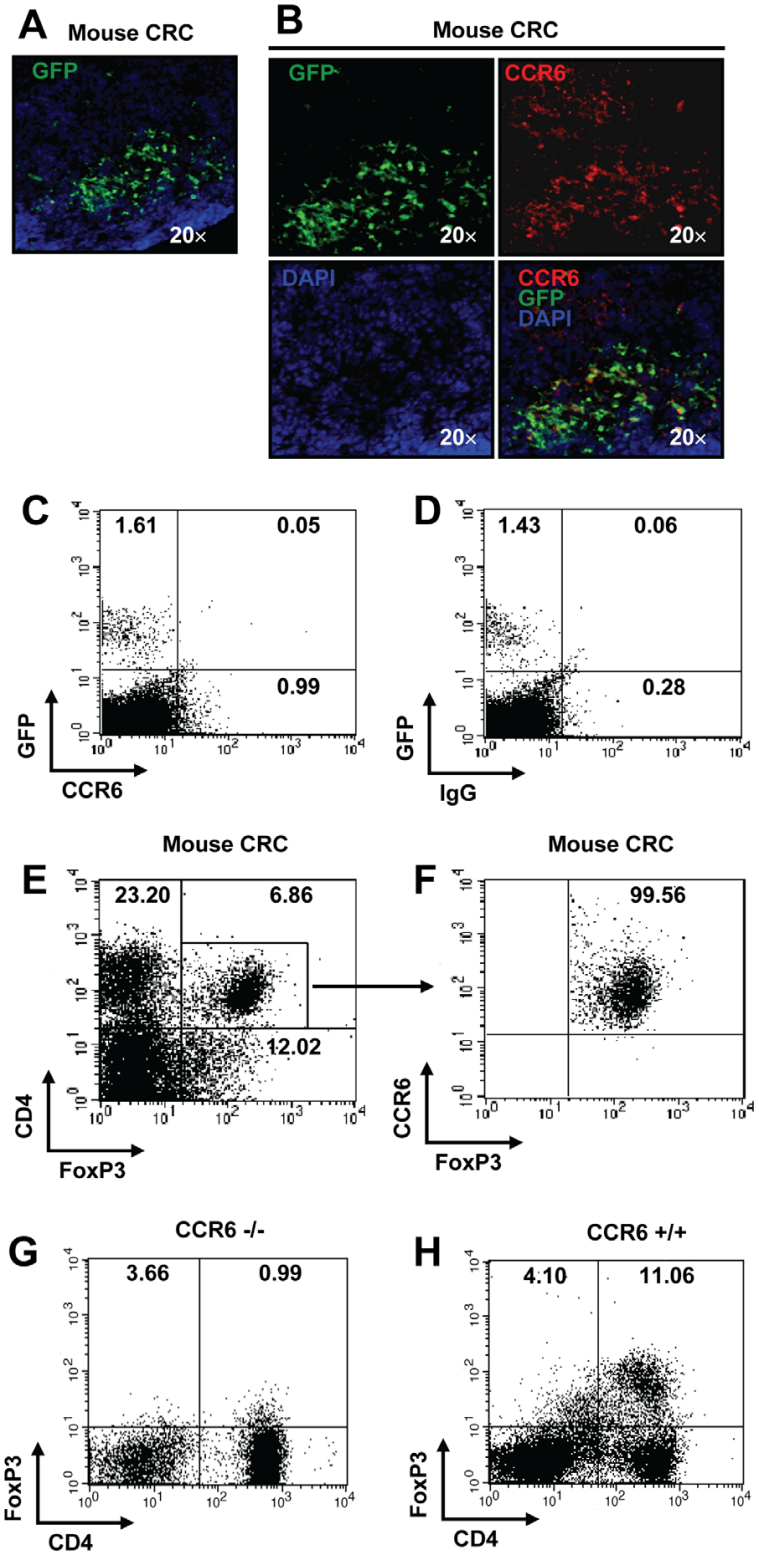
Requirement of CCR6 for trafficking of Treg-cells to CRC in mice. Treg-cells (0.5–1×10^6^) purified from spleens of FoxP3^GFP^ mice were adoptively transferred into mice bearing CRC induced by MNU and H. *pylori*. (**A**) Cryosections from tumor were stained for cell nuclei with DAPI to investigate infiltration of FoxP3^GFP+^ Treg-cells in tumor mass (original magnification, ×20). (**B**) Co-localization of GFP with CCR6 staining. (**C**, **D**) Flow cytometric analysis for CCR6 of FoxP^GFP+^ Treg-cells derived from peripheral blood of naïve FoxP3^GFP^ mice. (**E**, **F**) Flow cytometric analysis for CCR6 of infiltrating Treg-cells derived from CRC induced by MNU and H. *pylori* in mice. To confirm whether CCR6 is required for FoxP3^+^ Treg-cell trafficking, CMT93 cell line was grafted into CCR6−/− and CCR6+/+ mice. (**G**, **H**) Flow cytometric analysis of tumor-infiltrating CD4^+^FoxP3^+^ Treg-cells two weeks after CMT93 cell line grafting in CCR6−/− (**G**) and CCR6+/+ (**H**) mice. Representative data are shown which had been reproduced in 4 independent experiments.

To address the question of whether naturally occurring FoxP3^+^ Treg-cells are able to migrate to tumor tissues after adoptive transfer, we injected 0.5–1×10^6^ Treg-cells that were isolated from FoxP3^GFP^ transgenic mouse in to mice bearing CRC induced by MNU and H. *pylori* or healthy controls. Two weeks post adoptive transfer, cryosections derived from tumor tissue of mice injected with FoxP3^GFP+^ cells were examined for GFP^+^ Treg-cells. We observed that GFP^+^ Treg-cells massively accumulated within the tumor (**Figure 2A**). Notably, we identified that these accumulating GFP^+^ Treg-cells within colorectal tumor expressed CCR6, the Treg-cell homing receptor (**Figure 2B**). However, naïve Treg-cells derived from FoxP3^GFP^ transgenic mice did not express CCR6 (**Figure 2C–D**). In addition, we digested colorectal tumor from mice treated with MNU and H. *pylori*, and determined CCR6 expression of tumor-infiltrating Treg-cells by Flow cytomery. Consistently, we found tumor-infiltrating Treg-cells almost exclusively expressed CCR6 (**Figure 2E, F**). In order to confirm whether CCR6 was required for migration of Treg-cells to tumor sites, we grafted mouse colorectal tumor cell line CMT93 into CCR6 −/− mice. Two weeks after CMT93 grafting, tumor-infiltrating Treg-cells in CCR6 −/− mice were examined by Flow cytomery. We observed that the infiltration of Treg-cells was completely prevented in the absence of CCR6 in CCR6 −/− mice compared with CCR6 +/+ mice (**Figure 2G, H**). These data suggest that the homing and trafficking of tumor-infiltrating Treg-cells to the tumor mass is dependent on the chemokine receptor CCR6 *in vivo* in the CRC mouse model grafted with CMT93.

### TAMs contributed to the production of CCL20, the ligand of CCR6

**Figure 3:**
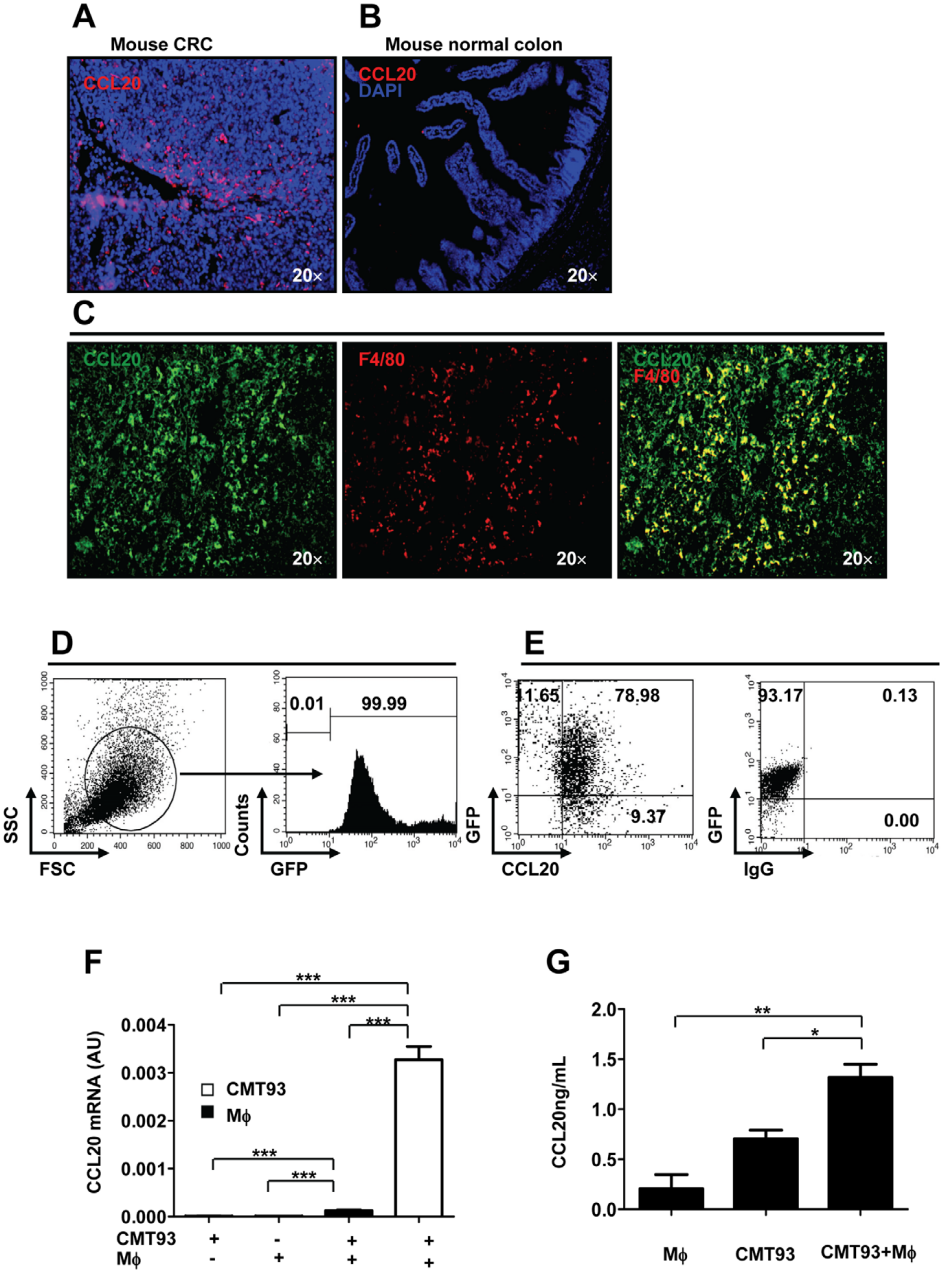
Contribution of TAMs to CCL20 production in CRC in mice. (**A**, **B**) Cryosections from mouse CRC induced by MNU and H. *pylori* or mouse normal colon were stained for CCL20 (red, original magni-fication, ×20). Double immunostaining with anti-mouse CCL20 and F4/80 mAb was performed on cryosections derived from mouse CRC. (**C**) Co-localization of CCL20 with F4/80 staining (original magnification, ×20). (**D**) CMT93 was transducted with GFP, and confirmed by flow cytometry. (**E**) Flow cytometric analysis of CCL20^+^ cells three weeks after GFP^+^ CMT93 cells grafting in C57BL/6J mice. (**F**) mRNA levels of CCL20 for co-culture of mouse peritoneal macrophages with CMT93 cancer cells. AU, arbitrary units. (**G**) Protein levels of CCL20 for co-culture of mouse peritoneal macrophages with CMT93 cancer cells. Representative data are shown which had been reproduced in at least 2 independent experiments. * *p*<0.05, ** *p*<0.01,*** *p*<0.001, Student's *t* test.

CCL20 is so far known to be the only ligand for CCR6 and capable of directing the migration of CCR6^+^ cells [20]. To examine the possible production of CCL20 in tumor tissue, tumor cryosections taken from C57BL/6J mice treated with MNU and H. *pylori* were immunostained with anti-mouse CCL20 mAb. We observed that expression of the chemokine CCL20 (red staining) was markedly increased in CRC tissues compared with normal mucosa (**Figure 3A, B**). Coordinated signaling between tumor cells and nonmalignant inflammatory cells in the tumor microenvironment is required for the development of tumors. Among them, macrophages are major producers of proinflammatory cytokines such as tumor necrosis factor-a (TNF-α), IL-1β, and IL-6, and play a crucial role in modulating immune responses [22]. In order to investigate whether macrophages are an important source of CCL20 in the tumor sites, double immunofluorescence staining was performed on cryo-sections derived from tumor biopsies of mice treated with MNU and H. *pylori*. We observed that in addition to F4/80^+^ macrophages, other cells also produced CCL20 in the tumor sites of mice (**Figure 3C**), indicating that CCL20 released by TAMs or others may induce Treg-cell migration to tumors. To clarify what other cells produce CCL20, we transducted CMT93 with GFP, and grafted subcutaneously to C57BL/6J mice (**Figure 3D**). Three weeks later, we detected the majority of CCL20^+^ cells (78.98%) was GFP^+^ CMT93 tumor cells, suggesting most of other CCL20 producing cells are tumor cells (**Figure 3E**). To determine whether macrophages are responsible for the overproduction of CCL20 in the tumor mass, we co-cultured CMT93 cancer cells with fresh mouse peritoneal macrophages for 48 hrs in a transwell apparatus. We demonstrated that in the co-culture both macrophages and CMT93 cancer cells produced increased levels of CCL20 compared with either macrophages or CMT93 culture alone (**Figure 3F, G**). Strikingly, in the co-culture of CMT93 and mouse peritoneal macrophages, CMT93 cancer cells produced 26.4-fold more CCL20 than macrophages at mRNA levels, suggesting that macrophages may interplay with cancer cells for production of CCL20 in the tumor microenvironment (**Figure 3F**). In addition, increased protein levels of CCL20 were confirmed by ELISA in the co-culture (**Figure 3H**).

### Simultaneous injection of recombinant mouse CCL20 results in the trafficking of CCR6^+^ Treg-cells into tumors, and promotion of CRC growth in mice

**Figure 4:**
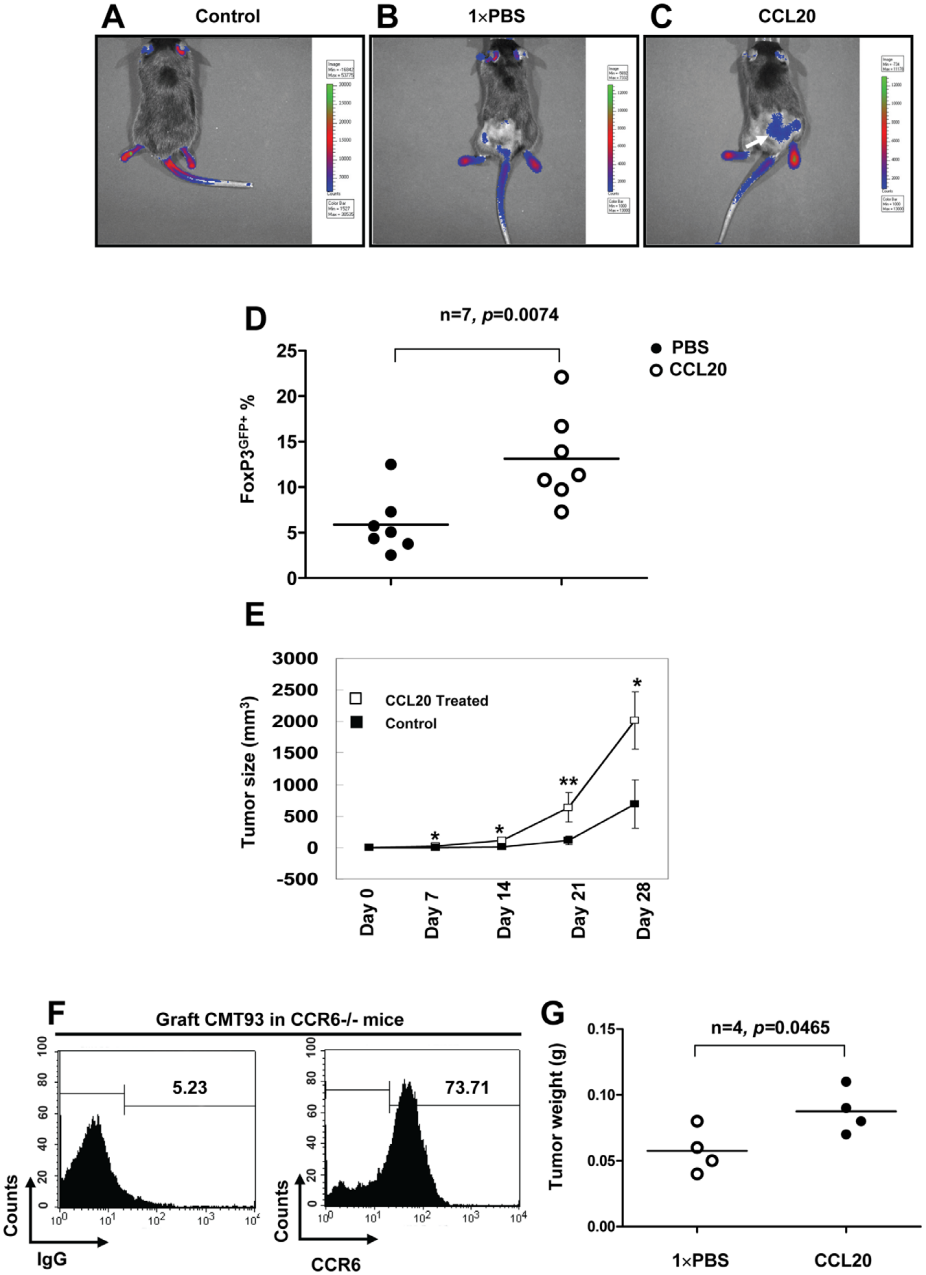
CCL20 recruited FoxP3^+^ Treg-cells to tumor mass and stimulated the growth of CRC in mice. Mouse colorectal cell line, CMT 93 (1×10^6^) with or without 0.5 µg mouse recombinant mouse CCL20 in 100 µl PBS were injected s.c. into FoxP3^GFP^ mice, and 0.5 µg recombinant mouse CCL20 in 100 µl PBS was injected s.c. weekly for 28 days. Control FoxP3^GFP^ mice were only given 100 µl PBS injection. (**A**) Normal FoxP3^GFP^ mouse was used as a technique control. (**B**, **C**) Green fluorescence of FoxP3^GFP+^ cells in mice treated PBS or recombinant mouse CCL20 (white arrow) were monitored using the IVIS Imaging System at day 28. (**D**) Numbers of tumor-infiltrating FoxP3^GFP+^ Treg-cells derived from grafted CRC in mice treated with PBS or recombinant mouse CCL20 were analyzed by Flow cytometry. (**E**) The sizes of tumors were measured with calipers at the indicated time points for 28 days. Injection of recombinant mouse CCL20 resulted in a significant increase in tumor sizes (n = 5). (**F**) Expression levels of CCR6 by grafted CRC in CCR6−/− mice. (**G**) CMT 93 (1×10^6^) with or without 0.5 µg mouse recombinant mouse CCL20 in 100 µl PBS were injected s.c. into CCR6−/− mice, and 0.5 µg recombinant mouse CCL20 in 100 µl PBS or 100 µl PBS was injected s.c. at every second day for 21 days. Tumor weight of CRC was measured at the end of experiment. Representative data are shown which had been reproduced in 3 independent experiments. * *p*<0.05, ** *p*<0.01, Student's *t* test.

Tumor-infiltrating Treg-cells suppress the priming and effector function of tumor killing effector T cells, contributing to tumor growth and development [11]. To determine the role of CCL20 in the migration of Treg-cells *in vivo*, we s.c. injected CMT93 murine CRC cells together with recombinant mouse CCL20 into FoxP3^GFP^ transgenic mice. Using the whole-body small animal fluorescence imaging system to monitor the migration of Treg-cells [23], we demonstrated that recombinant mouse CCL20 recruited a large number of GFP^+^ Treg-cells into tumors (white arrow) compared to PBS treated groups (**Figure 4A–C**). In order to further confirm GFP labeled Treg-cells migrated to the tumor mass in response to recombinant mouse CCL20, single cell suspensions of tumor tissue were prepared and FoxP3^GFP+^ Treg-cells were detected by Flow cytomery at the end of the experiment. In contrast to PBS treatment, we observed that GFP^+^ Treg-cells were significantly increased within the tumor that was treated with recombinant mouse CCL20 (**Figure 4D**). To further study the pathological importance of CCL20, we administrated recombinant mouse CCL20 around tumor sites weekly for 4 weeks. We showed that tumor size was significantly increased in mice treated with recombinant mouse CCL20 compared with PBS controls (**Figure 4E**), suggesting a critical role of CCL20 in CRC growth and development. In human CRC patients, CCR6 was also found to be expressed by primary CRC cells [24]. Therefore, for the definitive confirmatory study, we grafted CMT93 CRC cancer cells into CCR6−/− mice. In this model, tumor-infiltrating lymphocytes (TIL) will not express CCR6. Two weeks later we analyzed by Flow cytometry for CCR6 expression of single cell suspension prepared from graft CRC. Consistent with study in human CRC patients [24], we noted that in CCR6−/− mice, as much as 73.71% of graft CRC cells expressed CCR6 (**Figure 4F**). Most interestingly, we observed that recombinant mouse CCL20 stimulated CMT93 CRC cell growth by directly activating CCR6 on tumor cells in the absence of CCR6^+^ TIL in CCR6−/− mice (**Figure 4G**). This CCL20 mediated effect is likely to occur via attracting Treg-cells to the tumor mass or directly stimulating CRC cancer cells or both.

### Selective depletion of macrophages in CD11b-DTR mice decreased CCL20 tumor levels, blocked CCR6^+^ Treg-cell recruitment into the tumor mass and inhibited tumor growth

**Figure 5:**
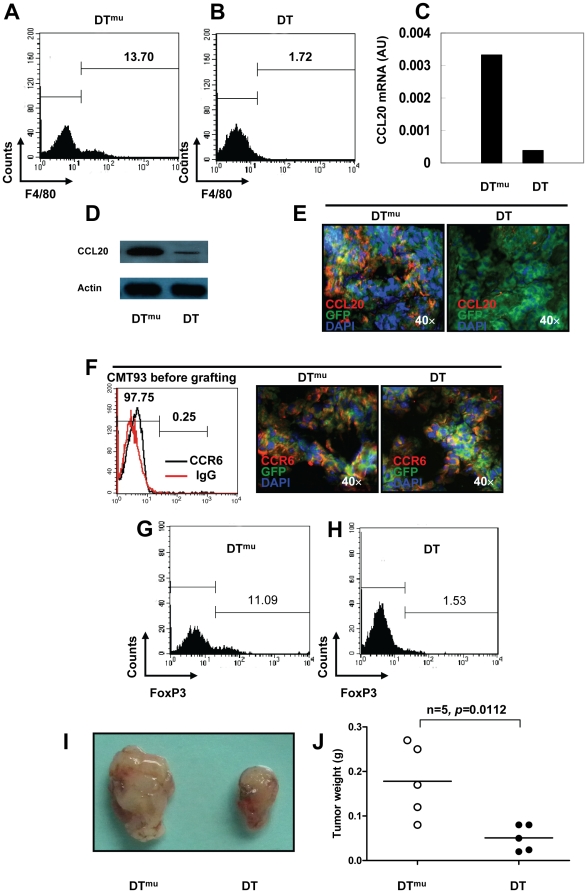
Conditional macrophage ablation disrupted Treg-cell recruitment and inhibited the growth of CRC in mice. (**A**, **B**) Flow cytometric analysis of TAMs derived from grafted CRC in CD11b-DTR mice injected with DT^mu^ or DT. (**C**) CCL20 mRNA levels in grafted CRC in CD11b-DTR mice injected with DT^mu^ or DT (data from 5 mice of each group were pooled together). (**D**) Lysates of grafted CRC from CD11b-DTR mice injected with DT^mu^ or DT were subjected to western blotting for CCL20 expression. Actin - loading control. (**E**) Immunofluorescence staining with anti-mouse CCL20 was performed on cryosections of GFP^+^ CMT93 grafted tumor mass derived from CD11b-DTR mice injected with DT^mu^ or DT for 14 days. (**F**) Flow cytometric analysis of CCR6 expression for GFP^+^ CMT93 cells before grafting (left panel), and CCR6 staining on cryosections of GFP^+^ CMT93 grafted tumor mass derived from CD11b-DTR mice injected with DT^mu^ or DT for 14 days (middle and right panel). (**G**, **H**) Number of tumor-infiltrating FoxP3^+^ Treg-cells in grafted CRC in CD11b-DTR mice injected with DT^mu^ or DT. (**I**) CRC grafted in CD11b-DTR mice injected with DT^mu^ or DT for 14 days. (**J**) Tumor weight of CRC grafted in CD11b-DTR mice injected with DT^mu^ or DT for 14 days. Representative data are shown which had been reproduced in 2 independent experiments.

CD11b-DTR transgenic mice have CD11b promoter-mediated expression of the human diphtheria toxin (DT) receptor, and this inducible system selectively depletes macrophages *in vivo* when DT is injected [25]. To examine whether *in vivo* DT treatment could eliminate TAMs in CMT93 graft CRC model, we first injected DT or DT^mu^ at a concentration of 25 ng/g mouse weight one day prior to grafting CMT93 in CD11b-DTR transgenic mice. Thereafter, we treated mice with DT or DT^mu^ at a concentration of 5 ng/g every three days for 14 days. In agreement with others, we demonstrated that DT selectively depleted F4/80^+^ macrophages 24 h after injection, but had no significant effect on T cells, neutrophils, mast cells or mature Langerhans cells/dendritic cells (data not shown) [26]. At the end of the experiment, our data revealed that DT treatment completely eliminated F4/80^+^ TAMs in CRC compared with DT^mu^ injection (**Figure 5A, B**). We further examined CCL20 expression in tumor tissue derived from mice treated with DT, we found that selective depletion of macrophages resulted into a marked decrease in both CCL20 mRNA level (**Figure 5C**) and CCL20 protein level (**Figure 5D**) in tumor mass compared with DT^mu^ treatment. To investigate whether TAMs promote the release of CCL20 from CMT93 tumor cells *in vivo*, we grafted GFP^+^ CMT93 tumor cells in CD11b-DTR transgenic mice. After treatment of DT or DT^mu^ for 14 days, we observed that the selective depletion of TAMs evidently inhibited the production of CCL20 by CMT93^GFP^ mouse tumor cells, suggesting that TAMs are not only the major source of CCL20 but also distinctly contribute to the release of CCL20 from CRC cells in mice (**Figure 5E**). Furthermore, we demonstrated that DT treatment did not decrease CCR6 expression by CMT93^GFP^ mouse tumor cells in comparison to DT^mu^ (**Figure 5F**). However, mouse CRC cells have low CCR6 expression before grafting to hosts (**Figure 5F**). Most importantly, macrophage depletion by administration of DT led to a marked reduction in the number of tumor-infiltrating FoxP3^+^ Treg-cells (**Figure 5G, H**). Together, these data indicate that TAMs support the accumulation of CCR6^+^ tumor-infiltrating Treg-cells, and, at least in part via this mechanism, promote the release of CCL20 in CRC in mice. Finally, we found that selective elimination of TAMs dramatically inhibited the growth of CRC in mice (**Figure 5I, J**), demonstrating a critical tumor-promoting role of TAMs in CRC mouse model.

## Discussion

The innate and adaptive immune systems can promote tumor development through inflammation-dependent mechanisms [2], [27]. This event is coordinated by many inflammatory cells, among them FoxP3^+^ Treg-cells are thought to suppress T cell immunity to tumor-associated antigens and to facilitate tumor progression [4]. FoxP3 is the most definitive maker of Treg-cells, and its detection could allow a better understanding of the mechanisms through which Treg-cells are involved in autoimmune and infectious diseases, as well as transplantation and tumor immunity [28]. Although many studies have focused on the role of Treg-cells in controlling self-reactivity, there is a growing body of evidence indicating that Treg-cells have a significant impact on the host response to cancer [29], [30], including CRC [31], [32]. It was previously reported that patients with high frequencies of infiltrating CD3^+^ T cells within their colorectal tumors have been thought to have a better 5-year survival rate (73%) than those with low levels of CD3^+^ T cells (30%) [33]. However, within this CD3^+^ T cell population, apart from effector T cells, Treg-cells are considered to suppress anti-tumor immune responses in patients with CRC [34]. In this study we have also confirmed that FoxP3^+^ Treg-cells are highly accumulated in the microenvironment in CRC mouse model, supporting the notion that the presence of Treg-cells may allow the escape of tumor cells from immune surveillance by cytotoxic T lymphocytes in CRC patients.

Like other human diseases, mouse models are widely used to elucidate mechanisms involved in colon carcinogenesis (initiation, promotion and progression) as well as studies on chemoprevention and intervention [35]. Herein, we induced CRC in C57BL/6J mice with MNU and H. *pylori* to investigate Treg-cell trafficking, differentiation and function. MNU is a direct alkylating agent that does not require metabolic activation and thus is a potent topical carcinogens [21]. We have found the strategy used for the induction of CRC in mice is quite effective as 85% mice used in this study developed visible CRC under the challenge of MNU and H. *pylori* (unpublished observations).

Evidence supporting an essential role for FoxP3^+^ Treg-cells in tumor immune evasion comes from studies that eliminated Treg-cells before tumor challenge. This treatment resulted in tumor rejection in several mouse tumor models [10], [36], [37], [38]. As in human settings [34], an increased numbers of Treg-cells were observed both in peripheral blood (**Figure S2**) and solid tumors in mice treated with MNU and H. *pylori* compared with normal colon of untreated mice. Most interestingly, in solid tumors derived from mice treated MNU and H. *pylori*, the frequency of CD4^+^FoxP3^+^ Treg-cells was an average of 9.095% of the total TIL, suggesting that these accumulated Treg-cells may serve as a common immune evasion mechanism to impede immunosurveillance against autologous tumor cells. Treg-cells perform an important function to limit autoimmunity and so targeting Treg-cell migration may serve as a novel and preferred approach in the immunotherapy of overall tumor progression. Additionally, we have detected some CD4^−^ cells expressing FoxP3 in the tumor mass. The origin of CD4^−^FoxP3^+^ cells will be submitted to the future investigation.

To function effectively in the tumor microenvironment, Treg-cells must migrate to and operate in various lymphoid and non-lymphoid organs. Similar to effector T cell populations, Treg-cell populations are subdivided on the basis of their expression of homing receptors into subsets that are predicted to migrate preferentially to certain tissues [39]. Studies with human ovarian cancers have revealed that Treg-cells home to the tumor mass and ascites *via* CCR4 in response to tumor cell- and TAM-derived CCL22 [15]. Yet, emerging evidence has suggested that apart from CCR4 some other chemokine receptors are involved in guiding Treg-cell trafficking in different types of tumors in both human and mice [17], [40]. Therefore, chemokine receptor expression patterns appear to be highly heterogeneous within the peripheral FoxP3^+^ Treg-cell population [11]. To date, no single homing receptor has been identified that is specifically expressed by Treg-cells. However, among CD4^+^ T cells, the chemokine receptor CCR6 is mainly expressed by Treg-cells in various inflammatory settings [41]. Similarly, the CCR6 ligand CCL20 is constitutively expressed by intestinal epithelial cells, and upregulated in epithelial cells during inflammation [42]. Consistent with this concept, our data demonstrated that naïve Treg-cells derived from FoxP3^GFP^ mice do not express CCR6, while FoxP3^GFP+^ Treg-cells predominantly expressed high levels of CCR6 in tumor tissue 2 weeks after adoptive transfer into tumor bearing mice. Our data provide strong evidence that these CCR6^+^ Treg-cells exhibit a prevalent memory phenotype, and clearly they might be tumor antigen experienced.

We have demonstrated a critical role of CCL20 in the recruitment of Treg-cells into tumor tissue by the injection recombinant mouse CCL20. The administration of recombinant mouse CCL20 resulted in a marked accumulation of CCR6^+^ Treg-cells in tumor sites as detected by both immunofluorescence staining and an *in vivo* fluorescence imaging. Most importantly, our finding revealed that the administration of recombinant mouse CCL20 led to a marked increase in tumor size in our CRC mouse model. Previous studies evaluating CCR6 expression levels by immunohistology in 64 CRC primary tumor specimens found that CCR6 staining was significantly stronger in cancer cells compared with adjacent colon epithelial cells [24]. In CCR6−/− mice grafted with CMT93, we convincingly showed that aside from TIL, 73.71% of CRC cells expressed CCR6. Using GFP labeled CMT93, we demonstrated that besides macrophages the majority of CCL20 producing cells in the tumor microenvironment were cancer cells. In this study, besides tumor-infiltrating CCR6^+^ Treg-cells we have confirmed the direct contribution of CCR6 expressed by CMT93 CRC cells to the CRC growth and development in CCR6−/− mice, the clinical application of targeting the CCR6-CCL20 chemokine axis may harbor therapeutic potential for the treatment of patients with CRC. However, our preliminary data indicated that the reconstitution of 10×10^6^ CD3^+^ T cells derived from FoxP3 knock-out mice (no FoxP3^+^ Treg-cells) resulted in a decrease in size of CRC in CCR6−/− mice compared with CD3^+^ T cells derived from wild type mice (unpublished observation), hints to promoting effect of CCR6^+^ Treg-cells in the development of CRC in mice.

Ablation of macrophage function, or inhibition of their infiltration into experimental tumors, inhibits growth and metastases, strongly suggests that macrophages are involved in promoting tumor progression [43]. The gastrointestinal mucosa is the largest reservoir of macrophages in the body. These important effector cells are derived from blood monocytes that are recruited to the lamina propria by inflammatory chemokines and bacterial products during inflammation [44]. Rectal tumors are usually infiltrated by TAMs and it is widely accepted that TAMs in various tumor tissues assist tumor growth by producing various growth factors and proangiogenic cytokines. The tumor environment is thought to ‘educate’ macrophages towards a tumor-promoting M2 phenotype [27], however the mechanisms underlying this phenomenon are not fully understood. Because macrophages interplay with tumor cells to produce a large amount of CCL20 in the co-culture, and the selective depletion of macrophages completely disrupted the release of CCL20 not only by TAMs but also mouse CRC cells in CD11b-DTR mice, we conclude that TAMs significantly contribute to the production of CCL20 which drives CCR6^+^ Treg-cell recruitment to the tumor tissue. These results indicate that the tumor microenvironment educates TAMs to switch to a specific phenotype for the production of a wide variety of growth factors, cytokines and chemokines that initiate and promote tumor progression [27].

Despite accumulating evidence indicates that high numbers of TAMs in the mass of solid tumors correlate with poor cancer prognosis [45], [46], results about the role of TAMs in tumorigenesis in CRC seem to be somewhat contradictory compared with other cancers. In a study including 131 CRC cases, macrophages positively influenced prognosis, although not significant in multivariate analysis [47], [48]. Furthermore, it was reported that low infiltration of macrophages tended to occur with more advanced CRC, and high macrophage infiltration along the tumor front correlates with improved survival indicating that TAMs may act as one cell population against malignant cells in patients with CRC [49], [50]. We here demonstrated for the first time that selective ablation of TAMs resulted in an inhibition of tumor progression, supporting a dominant protumorigenic role for TAMs in our mouse model of CRC.

In conclusion, our data has identified a previously unrecognized role for TAMs in contributing to the increased production of CCL20 that recruits CCR6^+^ Treg-cells and promotes CRC development in mice. This study highlights targeting CCL20 by down-regulating the activity of TAMs or its counter receptor CCR6 to block memory Treg-cell recruitment may present a promising strategy for the treatment of human CRC.

## Materials and Methods

### Ethics statement

Mice were kept under specific pathogen-free (SPF) conditions in compliance with the National Institutes of Health *Guide for the Care and Use of Laboratory Animals* with the approval (SYXK-2003-0026)of the Scientific Investigation Board of Shanghai Jiao Tong University School of Medicine, Shanghai, China. To ameliorate any suffering of mice observed throughout these experimental studies, mice were euthanized by CO_2_ inhalation. Clinical samples were obtained from Ruijin Hospital, Shanghai Jiao Tong University School of Medicine following protocols approved by Shanghai Jiao Tong University School of Medicine Review Board and following the Declaration of Helsinki Principles. PBMC or tissues from patients with colorectal cancer were collected simultaneously. Patients gave written informed consent. Sex and age-matched control samples were from normal volunteers.

### Mice

The C57BL/6J mice, B6.129P2-*Ccr6^tm1Dgen^*/J (designated as CCR6−/−) mice, B6.Cg-Foxp3^tm2Tch/J^ (designated as FoxP3^GFP^) mice, B6.FVB-Tg (ITGAM-DTR/EGFP) 34Lan/J (designated as CD11b-DTR) mice were purchased from the Jackson Laboratory (Bar Harbor, ME).

### Induced mouse CRC model

CRC was induced by N-methyl-N-nitrosourea (MNU) (Sigma Chemical) and H. *pylori* (SS1) in 89 pathogen-free, 6-week-old female C57BL/6J mice. In the first 10 weeks, MNU was dissolved in distilled water at the concentration of 240 ppm and was freshly prepared twice per week for administration in drinking water in light-shielded bottles. The treatment MNU was performed one week for administration and one week paused. During weeks 12 to 13, H. *pylori* was administrated intragastrically to the MNU treated mice at a concentration of 1×10^9^ C.F.U/ml, 0.15 ml per treatment, twice every other day. Apart from the treatment, the mice were fed with normal food for rest of the time until 80 weeks.

### Syngenic graft CRC model

Mouse colorectal cell line, CMT 93, was purchased from ATCC and maintained in DMEM supplemented with 10% fetal bovine, l-glutamine, 100 units of penicillin-G, and 100 µg/ml of streptomycin. CMT 93 (1×10^6^) with or without 0.5 µg recombinant mouse CCL20 in 100 µl 1×PBS were injected subcutaneously (s.c.) into FoxP3^GFP^ mice, and 0.5 µg recombinant mouse CCL20 in 100 µl 1×PBS was injected s.c. weekly for 21 or 28 days. Control FoxP3^GFP^ mice were only given 100 µl 1×PBS injection. Green fluorescence of FoxP3^GFP+^ cells was monitored using the IVIS Imaging System (Xenogen IVIS 200 System, Xenogen Inc., Hopkinton, MA, USA) at day 28. The size of the resulting tumors was measured with calipers weekly.

### GFP transduction

Lentivirus packaging system included pGC-FU-EGFP-IRES-Puromyciny, pMD2.G and psPAX2. DNA of the three plasmids was extracted using Endo-free Plasmid Mini Kit (Qiagen) and then used to transfect 293T cells using CaPO4 transfection reagents according to the manufacturer's instructions. 48 hours later, the supernatant was collected and centrifuge the supernatant 10 min at 2000×g, 4°C, then filter through a 0.45-µm pore-size filter. The flow-though is used directly for transduction of CMT93 cells. 48 hours later, puromycin was added at 5 ug/mL to select the CMT93 cells which stable express GFP. GFP^+^ CMT93 cells were further purified by Fluorescence Activated Cell Sorter, and expanded in the culture medium.

### CMT93 and macrophages

For co-culture experiments, a transwell apparatus with 3.0 µm pore size tissue culture treated polycarbonate membrane (24-well plate, Lot#34409007, Costar) was applied to cancer cell/macrophage co-cultures. Mouse peritoneal macrophages were prepared as published before [51]. 0.5×10^6^ CMT93 cancer cells were cultured with or without 0.5×10^6^ macrophages in 1 ml conditioned medium and incubated for 48 hrs. Total RNA was extracted from either CMT93 or macrophages for quantitative RT-PCR analysis of CCL20. Culture supernatant was collected to measure CCL20 protein levels using a Mouse CCL20/MIP-3 alpha Quantikine ELISA Kit (R&D, Cat. No.: MCC200). The details of conditioned medium preparation has been described previously [52].

### Depletion of macrophages

DT and the inactive form of the toxin DT-mutant (DT^mu^) were purchased from EMD Chemicals. CD11b-DTR mice (5 mice for each group) were given intraperitoneally (i.p.) either DT (25 ng/g) or DT^mu^ (25 ng/g), and 48 hours later CMT 93 (1×10^6^) in 100 µl 1×PBS were injected s.c. into CD11b-DTR mice treated with DT or DT^mu^. CD11b-DTR mice grafted with CTM93 were further treated with DT (5 ng/g) or DT^mu^ (5 ng/g) i.p. every 48 hours for 14 days. The actual tumor weight was measured at autopsy.

### Histological processing

Tissues from mice with CRC induced by MNU and H. *pylori* were fixed in 4% paraformaldehyde solution for 24 hours, and dehydrated, and embedded in paraffin. Paraffin-embedded 4 µm sections of CRC were stained with hematoxylin and eosin (H&E).

### Immunofluorescence staining

Frozen cryosections from mice with CRC were stained for immunological assessment of FoxP3 expression. Rat anti-mouse FoxP3 (eBioscience™, clone: FJK-16s) in conjugation with goat anti-rat Alexa Fluor®555 (invitrogen™), rat anti-mouse CCR6 (R&D System®, clone: MAB590) in conjugation with goat anti-rat Alexa Fluor®555 (invitrogen™), rat anti-mouse CCL20/MIP-3α (Santa Cruz Biotechnology) in conjugation with goat anti-rat Alexa Fluor®488 or goat anti-rat Alexa Fluor®555 (invitrogen™) or Alexa Fluor®555 (invitrogen™) and Alexa®555 conjugated rat anti-mouse F4/80 (Caltag Laboratories®, clone: CI:A3-1) were used. 4′,6-Diamidino-2-phenylindole (DAPI) (Fluka, Seelze, Germany) was employed to stain nuclei. Antibodies were diluted in a 1% antibody diluent (Dako®, Cat. No.: S3022). Isotype controls were used for all assays. Immunostainings were analyzed with a fluorescence microscope (Zeiss Axioskop 2 plus).

### FACS analysis

The following antibodies were used for flow cytometry: PE-conjugated anti-mouse FoxP3 staining kit (both from eBioscience™, San Diego, USA), APC-conjugated anti-mouse CD3 (eBioscience™ San Diego, USA), anti-mouse CCR6 (R&D System, clone: MAB590) in conjugation with goat anti-rat Alexa Fluor®555 (invitrogen™), APC-conjugated rat anti-mouse CD4 (BD Pharmingen™, clone: RM4-5), APC-conjugated anti-mouse CCR6 (BioLegend, clone: 29-2L17), and Alexa488-conjugated rat anti-mouse F4/80 (eBioscience, clone: BM8). Mouse colorectal tumor and normal colon tissues were digested by collagenase type I (Gibco, Grand Island,USA) and Hyaluronidase (Worthington, Lakewood, USA) for preparation of single cell suspension. Isotype controls were used for flow cytometric measurements.

### Quantitative RT-PCR

RNA extraction and PCR were carried out as previously described [53]. Primer sequences used were designed for murine CCL20 (5′-GACTGTTGCCTCTCGT-3′, 5′-TGACTCTTAGGCTGAGGA-3′). Cytokine and β-actin levels were calculated relative to the amounts found in a standard sample, and murine β-actin was employed for normalization as previously reported [53].

### Western blot analysis

Western blotting was performed as previously published [54]. Anti-mouse CCL20 mAb was purchased from R&D (Cat. Nr: MAB760, clone: 114906).

### Statistical analysis

Quantitative data is presented as mean values ± standard deviation (SD). Statistical significance was determined by means of the two-tailed Student's t test, or the Mann-Whitney U test in cases of a non-gaussian distribution. Differences were considered to be statistically significant at values of *p*<0.05 *, *p*<0.01 **, *p*<0.001 ***.

## Supporting Information

Figure S1 **Increased numbers of FoxP3^+^ Treg-cells in peripheral blood and tumor infiltrating lymphocytes of colorectal cancer patients.** Peripheral blood derived from either patients with CRC or healthy controls was processed for analysis of FoxP3 expression by FACS. (**A**, **B**) Numbers of Treg cells in peripheral blood of colorectal carcinoma patients (n = 8) and healthy individuals (n = 6). (**C**) A significant increase of numbers of FoxP3^+^ Treg cells was found in peripheral blood of colorectal carcinoma patients compared with healthy individuals (*p* = 0.008 by Student's t Test). (**D**) H&E staining of colorectal carcinoma derived from patients (original magnification, ×40). To examine tumor infiltrating Treg cells in patients with colorectal cancer, sections from paraffin-embed colorectal carcinoma tissue (n = 30) or human para-colorectal cancer tissue were subjected to indirect immunohistochemical staining of FoxP3. (**E**) Expression of FoxP3 (brown) in colorectal carcinoma (original magnification, ×20). (**F**) Higher magnification of (**E**) as indicated by the rectangle. (**G**) Negative expression of FoxP3 in para-colorectal cancer tissue (original magnification, ×20). (**H**) Higher magnification of (**G**) as indicated by the rectangle. Representative data are shown which had been reproduced in 4 independent experiments.
(TIF)

Figure S2 **Increased numbers of FoxP3^+^ Treg-cells in peripheral blood in mice with CRC.** Peripheral blood derived from either mice with CRC (n = 13) or normal controls (n = 8) was processed for analysis of FoxP3 expression by FACS. (**A**, **B**) Numbers of Treg-cells in peripheral blood of mice with CRC and normal control mice. (**E**) A significant increase of numbers of Treg-cells was observed in peripheral blood of mice with CRC compared with controls (*p* = 0.0219 by Student's t Test). Representative data are shown which had been reproduced in 3 independent experiments.
(TIF)

## References

[1] Kamangar F, Dores GM, Anderson WF (2006). Patterns of cancer incidence, mortality, and prevalence across five continents: defining priorities to reduce cancer disparities in different geographic regions of the world.. J Clin Oncol.

[2] Coussens LM, Werb Z (2002). Inflammation and cancer.. Nature.

[3] Fontenot JD, Gavin MA, Rudensky AY (2003). Foxp3 programs the development and function of CD4+CD25+ regulatory T cells.. Nat Immunol.

[4] Zou W (2006). Regulatory T cells, tumour immunity and immunotherapy.. Nat Rev Immunol.

[5] Ling KL, Pratap SE, Bates GJ, Singh B, Mortensen NJ (2007). Increased frequency of regulatory T cells in peripheral blood and tumour infiltrating lymphocytes in colorectal cancer patients.. Cancer Immun.

[6] Salama P, Phillips M, Grieu F, Morris M, Zeps N (2009). Tumor-infiltrating FOXP3+ T regulatory cells show strong prognostic significance in colorectal cancer.. J Clin Oncol.

[7] Heinze E, Baldwin S, Chan G, Hansen J, Song J (2009). Antibody-mediated FOXP3 protein therapy induces apoptosis in cancer cells in vitro and inhibits metastasis in vivo.. Int J Oncol.

[8] Valzasina B, Piconese S, Guiducci C, Colombo MP (2006). Tumor-induced expansion of regulatory T cells by conversion of CD4+CD25− lymphocytes is thymus and proliferation independent.. Cancer Res.

[9] Zhou G, Levitsky HI (2007). Natural regulatory T cells and de novo-induced regulatory T cells contribute independently to tumor-specific tolerance.. J Immunol.

[10] Ko K, Yamazaki S, Nakamura K, Nishioka T, Hirota K (2005). Treatment of advanced tumors with agonistic anti-GITR mAb and its effects on tumor-infiltrating Foxp3+CD25+CD4+ regulatory T cells.. J Exp Med.

[11] Qin FX (2009). Dynamic behavior and function of Foxp3+ regulatory T cells in tumor bearing host.. Cell Mol Immunol.

[12] van der Burg SH, Piersma SJ, de Jong A, van der Hulst JM, Kwappenberg KM (2007). Association of cervical cancer with the presence of CD4+ regulatory T cells specific for human papillomavirus antigens.. Proc Natl Acad Sci U S A.

[13] Vence L, Palucka AK, Fay JW, Ito T, Liu YJ (2007). Circulating tumor antigen-specific regulatory T cells in patients with metastatic melanoma.. Proc Natl Acad Sci U S A.

[14] Clark CE, Hingorani SR, Mick R, Combs C, Tuveson DA (2007). Dynamics of the immune reaction to pancreatic cancer from inception to invasion.. Cancer Res.

[15] Curiel TJ, Coukos G, Zou L, Alvarez X, Cheng P (2004). Specific recruitment of regulatory T cells in ovarian carcinoma fosters immune privilege and predicts reduced survival.. Nat Med.

[16] Sakaguchi S, Yamaguchi T, Nomura T, Ono M (2008). Regulatory T cells and immune tolerance.. Cell.

[17] Huehn J, Hamann A (2005). Homing to suppress: address codes for Treg migration.. Trends Immunol.

[18] Yoshida R, Imai T, Hieshima K, Kusuda J, Baba M (1997). Molecular cloning of a novel human CC chemokine EBI1-ligand chemokine that is a specific functional ligand for EBI1, CCR7.. J Biol Chem.

[19] Hromas R, Gray PW, Chantry D, Godiska R, Krathwohl M (1997). Cloning and characterization of exodus, a novel beta-chemokine.. Blood.

[20] Liao F, Rabin RL, Smith CS, Sharma G, Nutman TB (1999). CC-Chemokine Receptor 6 Is Expressed on Diverse Memory Subsets of T Cells and Determines Responsiveness to Macrophage Inflammatory Protein 3{alpha}.. J Immunol.

[21] Narisawa T, Magadia NE, Weisburger JH, Wynder EL (1974). Promoting effect of bile acids on colon carcinogenesis after intrarectal instillation of N-methyl-N′-nitro-N-nitrosoguanidine in rats.. J Natl Cancer Inst.

[22] Wang H, Peters T, Sindrilaru A, Scharffetter-Kochanek K (2009). Key role of macrophages in the pathogenesis of CD18 hypomorphic murine model of psoriasis.. J Invest Dermatol.

[23] Kim M-H, Liu W, Borjesson DL, Curry F-RE, Miller LS (2008). Dynamics of Neutrophil Infiltration during Cutaneous Wound Healing and Infection Using Fluorescence Imaging.. J Invest Dermatol.

[24] Ghadjar P, Coupland SE, Na I-K, Noutsias M, Letsch A (2006). Chemokine Receptor CCR6 Expression Level and Liver Metastases in Colorectal Cancer.. Journal of Clinical Oncology.

[25] Stoneman V, Braganza D, Figg N, Mercer J, Lang R (2007). Monocyte/Macrophage Suppression in CD11b Diphtheria Toxin Receptor Transgenic Mice Differentially Affects Atherogenesis and Established Plaques.. Circ Res.

[26] Duffield JS, Forbes SJ, Constandinou CM, Clay S, Partolina M (2005). Selective depletion of macrophages reveals distinct, opposing roles during liver injury and repair.. The Journal of Clinical Investigation.

[27] Pollard JW (2004). Tumour-educated macrophages promote tumour progression and metastasis.. Nat Rev Cancer.

[28] Roncador G, Garcia JF, Maestre L, Lucas E, Menarguez J (2005). FOXP3, a selective marker for a subset of adult T-cell leukaemia/lymphoma.. Leukemia.

[29] Jones E, Dahm-Vicker M, Golgher D, Gallimore A (2003). CD25+ regulatory T cells and tumor immunity.. Immunol Lett.

[30] Antony PA, Restifo NP (2002). Do CD4+ CD25+ immunoregulatory T cells hinder tumor immunotherapy?. J Immunother.

[31] Yaqub S, Henjum K, Mahic M, Jahnsen FL, Aandahl EM (2008). Regulatory T cells in colorectal cancer patients suppress anti-tumor immune activity in a COX-2 dependent manner.. Cancer Immunol Immunother.

[32] Loddenkemper C, Schernus M, Noutsias M, Stein H, Thiel E (2006). In situ analysis of FOXP3+ regulatory T cells in human colorectal cancer.. J Transl Med.

[33] Galon J, Costes A, Sanchez-Cabo F, Kirilovsky A, Mlecnik B (2006). Type, density, and location of immune cells within human colorectal tumors predict clinical outcome.. Science.

[34] Clarke SL, Betts GJ, Plant A, Wright KL, El-Shanawany TM (2006). CD4+CD25+FOXP3+ regulatory T cells suppress anti-tumor immune responses in patients with colorectal cancer.. PLoS One.

[35] Rosenberg DW, Giardina C, Tanaka T (2009). Mouse models for the study of colon carcinogenesis.. Carcinogenesis.

[36] Onizuka S, Tawara I, Shimizu J, Sakaguchi S, Fujita T (1999). Tumor rejection by in vivo administration of anti-CD25 (interleukin-2 receptor alpha) monoclonal antibody.. Cancer Res.

[37] Shimizu J, Yamazaki S, Sakaguchi S (1999). Induction of tumor immunity by removing CD25+CD4+ T cells: a common basis between tumor immunity and autoimmunity.. J Immunol.

[38] Sutmuller RP, van Duivenvoorde LM, van Elsas A, Schumacher TN, Wildenberg ME (2001). Synergism of cytotoxic T lymphocyte-associated antigen 4 blockade and depletion of CD25(+) regulatory T cells in antitumor therapy reveals alternative pathways for suppression of autoreactive cytotoxic T lymphocyte responses.. J Exp Med.

[39] Huehn J, Siegmund K, Lehmann JC, Siewert C, Haubold U (2004). Developmental stage, phenotype, and migration distinguish naive- and effector/memory-like CD4+ regulatory T cells.. J Exp Med.

[40] Campbell DJ, Ziegler SF (2007). FOXP3 modifies the phenotypic and functional properties of regulatory T cells.. Nat Rev Immunol.

[41] Kleinewietfeld M, Puentes F, Borsellino G, Battistini L, Rotzschke O (2005). CCR6 expression defines regulatory effector/memory-like cells within the CD25(+)CD4+ T-cell subset.. Blood.

[42] Kunkel EJ, Campbell DJ, Butcher EC (2003). Chemokines in lymphocyte trafficking and intestinal immunity.. Microcirculation.

[43] Lin EY, Nguyen AV, Russell RG, Pollard JW (2001). Colony-stimulating factor 1 promotes progression of mammary tumors to malignancy.. J Exp Med.

[44] Smith PD, Ochsenbauer-Jambor C, Smythies LE (2005). Intestinal macrophages: unique effector cells of the innate immune system.. Immunol Rev.

[45] Mantovani A, Sica A, Allavena P, Garlanda C, Locati M (2009). Tumor-associated macrophages and the related myeloid-derived suppressor cells as a paradigm of the diversity of macrophage activation.. Human Immunology.

[46] Leek RD, Lewis CE, Whitehouse R, Greenall M, Clarke J (1996). Association of macrophage infiltration with angiogenesis and prognosis in invasive breast carcinoma.. Cancer Res.

[47] Khorana AA, Ryan CK, Cox C, Eberly S, Sahasrabudhe DM (2003). Vascular endothelial growth factor, CD68, and epidermal growth factor receptor expression and survival in patients with Stage II and Stage III colon carcinoma: a role for the host response in prognosis.. Cancer.

[48] Funada Y, Noguchi T, Kikuchi R, Takeno S, Uchida Y (2003). Prognostic significance of CD8+ T cell and macrophage peritumoral infiltration in colorectal cancer.. Oncol Rep.

[49] Nakayama Y, Nagashima N, Minagawa N, Inoue Y, Katsuki T (2002). Relationships between tumor-associated macrophages and clinicopathological factors in patients with colorectal cancer.. Anticancer Res.

[50] Forssell J, Oberg A, Henriksson ML, Stenling R, Jung A (2007). High macrophage infiltration along the tumor front correlates with improved survival in colon cancer.. Clin Cancer Res.

[51] Li Y, Gerbod-Giannone M-C, Seitz H, Cui D, Thorp E (2006). Cholesterol-induced Apoptotic Macrophages Elicit an Inflammatory Response in Phagocytes, Which Is Partially Attenuated by the Mer Receptor.. Journal of Biological Chemistry.

[52] Chen JJW, Yao P-L, Yuan A, Hong T-M, Shun C-T (2003). Up-Regulation of Tumor Interleukin-8 Expression by Infiltrating Macrophages.. Clinical Cancer Research.

[53] Hedrick MN, Lonsdorf AS, Shirakawa AK, Richard Lee CC, Liao F (2009). CCR6 is required for IL-23-induced psoriasis-like inflammation in mice.. J Clin Invest.

[54] Li Q, Laumonnier Y, Syrovets T, Simmet T (2007). Plasmin Triggers Cytokine Induction in Human Monocyte-Derived Macrophages.. Arterioscler Thromb Vasc Biol.

