# Supplementary figures and images for: Tumor-Associated Macrophages Recruit CCR6+ Regulatory T Cells and Promote the Development of Colorectal Cancer via Enhancing CCL20 Production in Mice
Source: PLoS One. 2011 Apr 29;6(4):e19495. doi: 10.1371/journal.pone.0019495 (PMC3084880; doi:10.1371/journal.pone.0019495)

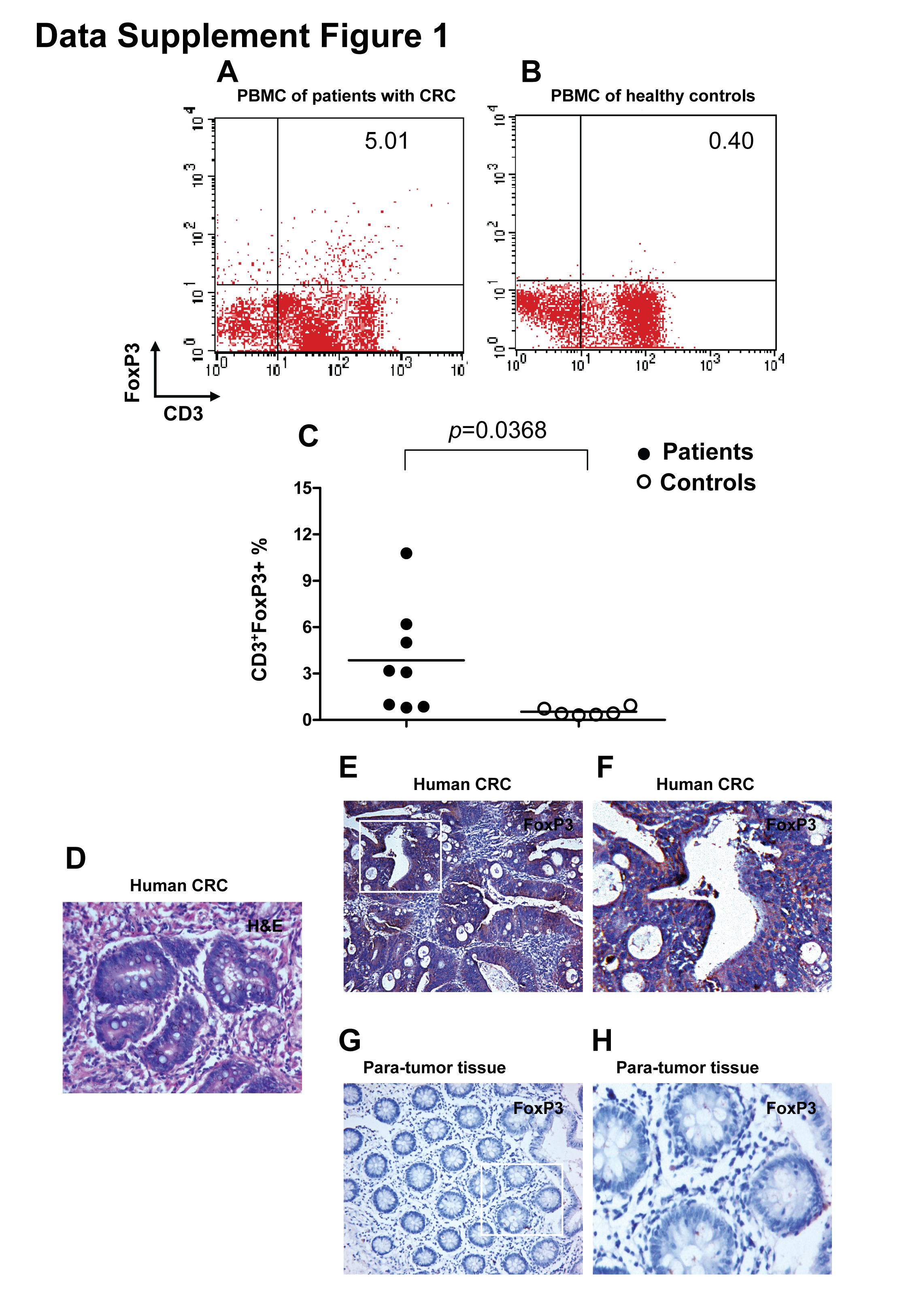

Supplement: Figure S1 — Increased numbers of FoxP3+ Treg-cells in peripheral blood and tumor infiltrating lymphocytes of colorectal cancer patients. Peripheral blood derived from either patients with CRC or healthy controls was processed for analysis of FoxP3 expression by FACS. (A, B) Numbers of Treg cells in peripheral blood of colorectal carcinoma patients (n = 8) and healthy individuals (n = 6). (C) A significant increase of numbers of FoxP3+ Treg cells was found in peripheral blood of colorectal carcinoma patients compared with healthy individuals (p = 0.008 by Student's t Test). (D) H&E staining of colorectal carcinoma derived from patients (original magnification, ×40). To examine tumor infiltrating Treg cells in patients with colorectal cancer, sections from paraffin-embed colorectal carcinoma tissue (n = 30) or human para-colorectal cancer tissue were subjected to indirect immunohistochemical staining of FoxP3. (E) Expression of FoxP3 (brown) in colorectal carcinoma (original magnification, ×20). (F) Higher magnification of (E) as indicated by the rectangle. (G) Negative expression of FoxP3 in para-colorectal cancer tissue (original magnification, ×20). (H) Higher magnification of (G) as indicated by the rectangle. Representative data are shown which had been reproduced in 4 independent experiments. (TIF) [file pone.0019495.s001.tif]

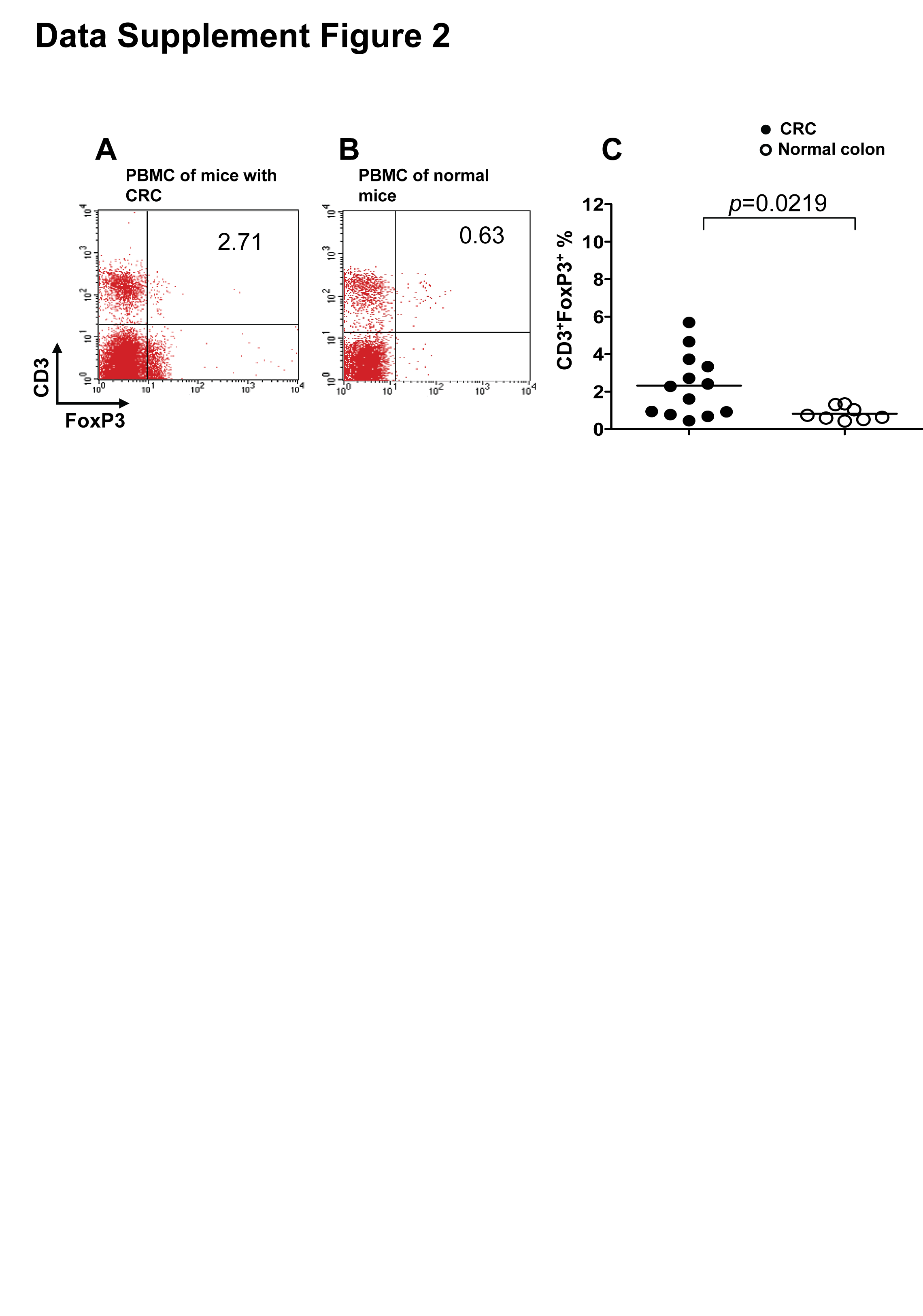

Supplement: Figure S2 — Increased numbers of FoxP3+ Treg-cells in peripheral blood in mice with CRC. Peripheral blood derived from either mice with CRC (n = 13) or normal controls (n = 8) was processed for analysis of FoxP3 expression by FACS. (A, B) Numbers of Treg-cells in peripheral blood of mice with CRC and normal control mice. (E) A significant increase of numbers of Treg-cells was observed in peripheral blood of mice with CRC compared with controls (p = 0.0219 by Student's t Test). Representative data are shown which had been reproduced in 3 independent experiments. (TIF) [file pone.0019495.s002.tif]
